# Mitochondrial NME6: A Paradigm Change within the NME/NDP Kinase Protein Family?

**DOI:** 10.3390/cells13151278

**Published:** 2024-07-30

**Authors:** Bastien Proust, Maja Herak Bosnar, Helena Ćetković, Malgorzata Tokarska-Schlattner, Uwe Schlattner

**Affiliations:** 1 Division of Molecular Medicine, Ruđer Bošković Institute, 10000 Zagreb, Croatia; bproust@irb.hr; 2 Division of Molecular Biology, Ruđer Bošković Institute, 10000 Zagreb, Croatia; cetkovic@irb.hr; 3 Univ. Grenoble Alpes, Inserm U1055, Lab. of Fundamental and Applied Bioenergetics (LBFA), 38058 Grenoble, France; malgorzata.tokarska-schlattner@univ-grenoble-alpes.fr; 4 Institut Universitaire de France (IUF), 75231 Paris, France

**Keywords:** NME, nucleoside diphosphate kinase, NM23, mitochondria, pyrimidine nucleotides, RCC1L, mtDNA, mtRNA

## Abstract

Eukaryotic NMEs/NDP kinases are a family of 10 multifunctional proteins that occur in different cellular compartments and interact with various cellular components (proteins, membranes, and DNA). In contrast to the well-studied Group I NMEs (NME1–4), little is known about the more divergent Group II NMEs (NME5–9). Three recent publications now shed new light on NME6. First, NME6 is a third mitochondrial NME, largely localized in the matrix space, associated with the mitochondrial inner membrane. Second, while its monomeric form is inactive, NME6 gains NDP kinase activity through interaction with mitochondrial RCC1L. This challenges the current notion that mammalian NMEs require the formation of hexamers to become active. The formation of complexes between NME6 and RCC1L, likely heterodimers, seemingly obviates the necessity for hexamer formation, stabilizing a NDP kinase-competent conformation. Third, NME6 is involved in mitochondrial gene maintenance and expression by providing (d)NTPs for replication and transcription (in particular the pyrimidine nucleotides) and by a less characterized mechanism that supports mitoribosome function. This review offers an overview of NME evolution and structure and highlights the new insight into NME6. The new findings position NME6 as the most comprehensively studied protein in NME Group II and may even suggest it as a new paradigm for related family members.

## 1. The NME Protein Family

NME (non-metastatic) proteins, also known as NM23 proteins or NDPKs (nucleoside diphosphate kinases), constitute a protein family conserved from bacteria to humans, with 10 known members in mammals [1,2,3]. Initially identified as exclusive “house-keeping” NDPKs responsible for the global supply of cellular (d)NTPs, it became evident that this family is functionally diverse, with many members being bi- or multi-functional. Already, their NDP kinase activity serves distinct functions, such as locally providing GTP to fuel GTPases or G-proteins [4,5]. Beyond this, NME proteins exhibit a range of additional functions, including protein histidine kinase activity [6,7], involvement in DNA transcription and repair [8], and the binding and transfer of phospholipids [9,10,11,12,13]. More recently, some NMEs were identified as major binders of (acyl)CoAs [14,15,16,17] and, thus, implicated in the transport and availability of cellular (acyl)CoAs [17,18]. These functions make NMEs multi-substrate enzymes and putative metabolic sensors. Likely, several of these mechanisms contribute to the metastasis suppressor function of certain NMEs, in particular NME1 and NME4 [19,20]. In eukaryotes, NME proteins are distributed across various cellular compartments, including the cytosol, mitochondria, peroxisomes, and nucleus, where they interact with other proteins, membranes, or DNA. The specific role of each particular NME is determined by its primary function in a specific subcellular location and/or interaction with specific partners [2,3].

## 2. Evolution within the NME Family

Human NMEs are divided into two groups based on phylogenetics, protein domains, and exon/intron structure [1]. Most of the prior research has centered around NME Group I (NME1–4), characterized by high amino acid sequence homology and a single NDP kinase domain. These proteins exclusively form hexamers, exhibit diverse cellular localizations, and display ubiquitous tissue expression. In contrast, the understudied Group II proteins (NME5–9) show less homology and feature single or multiple NDP kinase domains, often accompanied by extra-domains. However, evidence supporting oligomer formation and/or NDP kinase activity within Group II is limited. Bioinformatics analyses suggest the emergence of Group II NMEs early in eukaryote evolution [1], with ancestral-type Group II proteins conserved across three out of six major eukaryotic supergroups [21]. Likely, NME5- and NME7-like proteins were already present in the ancestor of all eukaryotes and resembled the red alga *Chondrus crispus* NME5 (NME5-likeCc). This ancient NME5-like protein demonstrates a multimeric structure and NDP kinase activity comparable to human NME1/2 proteins [21]. The NME6-like gene appeared by duplication of the NME5-like gene in the ancestor of the unikonts before the amoebozoan split [21]. An ancient NME6-like variant from the marine sponge *Suberites domuncula* (NME6Sd) seems to be devoid of NDP kinase activity [22]. Although it has a putative mitochondrial targeting sequence, it fails to localize to mitochondria when transfected into human cells. NME6Sd also differs from human NME6 by different transcriptional binding sites in the promoter region and the lack of two recent introns [22].

Collectively, these findings support the prevailing notion that NDP kinase activity depends on the assembly of NME into oligomers [23]. Unlike bacteria, where homooligomers exist as tetramers or hexamers, eukaryotes exclusively harbor hexameric complexes. The latter may represent homohexamers, but they are likely mostly heterohexamers consisting of different cytosolic NMEs (NME1, NME2, or likely NME3) [24,25]. Irrespective of the specific oligomerization state, all NME complexes share a fundamental structural unit, a dimer formed by the assembly of two monomers in a head-to-tail configuration [23]. Thus, oligomerization seems to be an evolutionarily conserved and pivotal feature crucial for the optimal functioning of NME proteins.

## 3. Early Data on NME6

NME6 was first described independently by the groups Lambeth [26] and Nakamura [27] in 1999. Although the NDK (nucleoside diphosphate kinase) domain comprises most of its amino acid sequence, as in Group I NMEs, NME6 represents a quite distinct NME isoform. Its overall amino acid identity is only about 30% compared with Group I members, and it has several amino acid (aa) insertions [28]: at the N terminus (7 aa), the C terminus (22 aa), position L30 (1 aa), and most importantly in the Kpn loop (3 aa), located close to the active site and involved in surface contacts that trigger protein oligomerization. Despite this relatively low sequence conservation, the overall protein fold of NME6 is highly conserved across both paralogues and orthologues [23]. In particular, the catalytic pocket of NME6 remains largely unchanged, conserving all the amino acids essential for the NDPK activity of NME1/2 [1].

**Figure 1:**
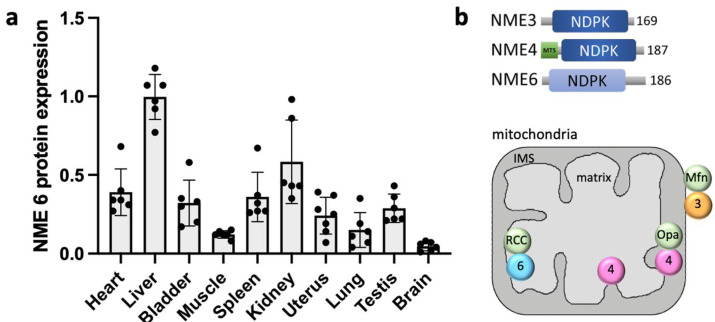
NME6 protein expression and overview of mitochondrial NMEs. (**a**) Quantification of NME6 relative abundance based on immunoblot data of NME6 in mouse tissues (strain C57BL/6J) using detection with a specific anti-NME6 primary antibody (HPA017909, Merck-Sigma-Aldrich, Burlington, MA, USA, dilution 1:1000). Data are normalized to Ponceau stain, with liver set to 1, showing means ± SEM (n = 6). (**b**) Overview of domain structure, localization, and interaction partners of mitochondrial NMEs (modified from [3,37]). Abbreviations: MTS, mitochondrial targeting sequence; 3, NME3; 4; NME4; 6, NME6; Mfn, mitofusin; Opa1, optic atrophy 1; RCC (RCC1L or WBSCR16), regulator of chromosome condensation 1-like. Mfn and Opa1 are dynamin-related GTPases.

Another remarkable characteristic of NME6 is its ubiquitous expression across mammalian tissues, as evidenced by its presence at both the RNA [26,27] and protein levels (Figure 1a). This feature is shared with Group I NMEs but sets it apart from other members of Group II. Elevated NME6 mRNA levels have been detected in numerous human tissues, including the heart, skeletal muscle, spleen, kidney, pancreas, placenta, testis, and ovary [27]. NME6 protein exhibits particularly high abundance in mice, specifically in the liver and kidney (Figure 1a).

The cellular function(s) of NME6 have long remained enigmatic, with only a few data points emerging regarding its potential involvement in human disease. Data from the Cancer Genome Atlas (https://portal.gdc.cancer.gov/; accessed on 29 July 2024) indicate increased NME6 gene expression in various cancer tissues, particularly liver hepatocellular carcinoma. Several studies have reported upregulated NME6 mRNA in tumor tissue, with some cases linking it to a negative prognosis, including in colorectal [29,30] and liver cancer [31,32,33]. Moreover, analyses from publicly available databases suggest a potential association between NME6 functions and cancer progression [34]. For instance, CRISPR KO screens indicate an intrinsic dependency on NME6 expression in many cancer cells. Clinically, elevated NME6 expression significantly correlates with poorer patient outcomes in several tumor types, most notably again in liver hepatocellular carcinoma [34].

NME6 may be involved in further health-relevant functions. First, the protein plays a role in the inflammatory response. NME6, along with NME3 and NME4, is a positive regulator of non-canonical inflammasome activation in mouse macrophages [35]. In particular, silencing NME6 significantly decreases IL-1α and IL-1β responses to lipopolysaccharide [35]. Further, NME6 appears to be essential for the renewal of embryonic stem cells, as its silencing triggers stem cell differentiation and reduces teratoma formation [36]. Importantly, homozygous NME6 knockout in mice is associated with embryonic lethality at early stages of development, suggesting it is an essential gene (International Mouse Phenotyping Consortium; MGI: 1861676).

## 4. NME6 as a New Key Player in Mitochondrial Gene Expression

While there has been a very limited amount of research on NME6 since its discovery 25 years ago, three noteworthy publications since 2021 have significantly enhanced our comprehension of NME6. Here, we summarize the key findings and implications derived from these recent studies carried out by our teams [37] and the groups led by T. MacVicar [38] and L. S. Churchman [39].

### 4.1. NME6 Is Predominantly an Inner Membrane-Associated Mitochondrial Matrix Protein

The mitochondrial localization of NME6, initially proposed by Tsuiki et al. [27], was conclusively validated through confocal microscopy and cell fractionation by us [37] and further corroborated by others [38,39]. This localization is also consistent with earlier large-scale proteomics studies [40,41,42]. Subsequent mitochondrial subfractionation [37] and proteinase K protection assays [39] precisely positioned NME6 within the mitochondrial matrix, in close proximity to, or associated with the inner mitochondrial membrane.

Consequently, three NMEs have now been localized to mammalian mitochondria (Figure 1b). The first, NME3, is anchored to the cytosolic side of the outer mitochondrial membrane by a hydrophobic domain and phosphatidic acid [13]. In this location, it interacts with the GTPases Mfn [43] and Drp1/Dnm1 [11,44], playing a crucial role in mitochondrial fusion and mitophagy. Notably, NME3 is also found in the cytosol, where it can bind to and function on other membranes, including the peroxisomal membrane [12] and the plasma membrane [25]. The second mitochondrial isoform, NME4, is fully imported into the mitochondrial intermembrane space (IMS) and the matrix through an N-terminal mitochondrial targeting sequence (MTS). It primarily interacts with cardiolipin in the inner membrane via its basic amino acids, as well as with the GTPase Opa1 [9,10]. Within the IMS, NME4 is involved in mitochondrial fusion, intermembrane phospholipid transfer, and mitophagy [4,45]. Finally, NME6 is imported into the matrix without a conventional MTS [46,47], suggesting the participation of a non-canonical mitochondrial import pathway.

### 4.2. NME6 Interaction with RCC1L Is Required for NDP Kinase Activity

**Figure 2:**
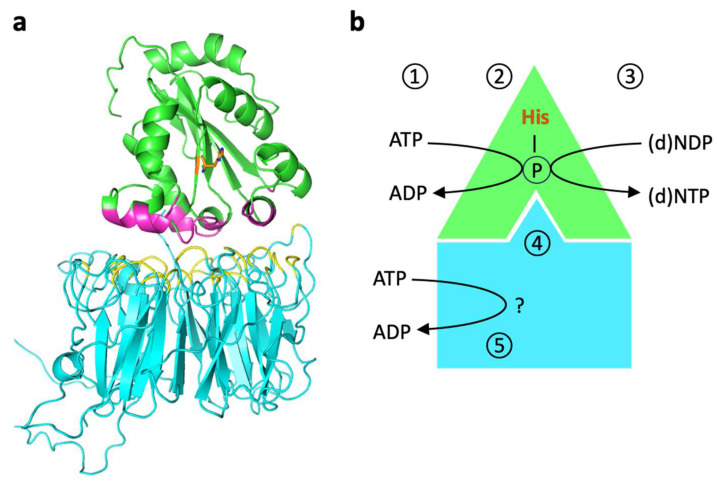
Putative structure and function of NME6-RCC1L complexes. (**a**) Model of the putative heterodimer structure. Interaction of NME6 (green, Alphafold model) and RCC1L (blue, PDB 5XG8 [54]), modeled by the AlphaFold_advanced ColabFold sheet. The interaction score is above 85%. The interacting amino acids of NME6 and RCC1L are shown in magenta and yellow, respectively. (**b**) Model of NME6/RCC1L functions. Within the heterodimer, NME6 catalyzes the classical, sequential, two-step ping-pong reaction that is different from most other ATP-dependent phosphorylations. This reaction transfers the γ-phosphate of any (d)NTP (physiologically mostly ATP) onto the nucleophilic histidine in the enzyme’s active site (1) to generate a phosphohistidine intermediate (2). Then, this phosphate is transferred onto the β-phosphate of any other (d)NDP binding subsequently to the active site (3). This finally yields all (d)NTPs needed, as, e.g., building blocks for mtRNA/DNA synthesis or sources of free energy for endergonic enzyme reactions. RCC1L renders NME6 NDP kinase-competent, probably by inducing an appropriate NME6 conformation (4). An endogenous ATPase activity of RCC1L may be involved in one of these or other processes (5) [39].

Among the two NME6 protein variants derived from the single *nme6* gene, we found the shorter 186 aa species to be overwhelmingly predominant [37]. Intriguingly, when this NME6 variant was expressed and purified from bacteria, it remained monomeric and did not engage in the formation of heterooligomers with other NME isoforms in vitro. Consistent with the above-mentioned notion linking NDPK activity to an oligomeric NME structure [23], these NME6 monomers exhibited no discernible NDPK activity with ATP and dTDP used as phosphate donor and acceptor, respectively, for the NDPK reaction [37,39] (see Figure 2). The presence of NDPK activity within NME6 has been somewhat controversial since its discovery. With recombinant NME6, Tsuiki et al. reported autophosphorylation with ^32^P-ATP and ^32^P-transfer to CDP, albeit at an exceedingly low rate [27]. However, Mehus et al. could not detect NDPK activity [26], and Yoon and colleagues also failed to observe ^32^P-ATP autophosphorylation or NDPK activity with ATP and dTDP [48].

In search of alternative functions for NME6, we compared large-scale unbiased interactomics/proteomics studies that targeted the mitochondrial matrix [41,42,49,50,51,52,53]. These studies consistently yielded RCC1-like G exchanging factor-like protein (RCC1L), also known as Williams-Beuren syndrome chromosomal region 16 protein (WBSCR16), as a potential interaction partner of NME6. Using proximity ligation assays and immunoprecipitation, we could indeed confirm the presence of stable NME6/RCC1L complexes in MDA-MB-231T cells [37]. The subsequent investigations into NME6 [38,39] not only validated the existence of such NME6/RCC1L complexes, which are likely heterodimers according to docking studies (Figure 2a), but also demonstrated that endogenous mammalian NME6 is NDPK active within these complexes. Specifically, NME6-KO cells showed a deficiency in the generation of pyrimidine NTPs, in particular CTP, which can be reversed by re-expressing wild-type NME6, but not a kinase-inactive NME6 variant lacking the catalytic histidine (Table 1) [38].

Reevaluation of the NDPK activity using recombinant NME6 and ^32^P-ATP autoradiography in vitro confirmed that NME6 alone is inactive, but while bound to RCC1L, it is able to transfer phosphate from ATP to both UDP and CDP [39]. In addition to this functional dependence of NME6, the stability of either protein seems to depend on the presence of the other. In particular, depletion of RCC1L results in a concomitant strong depletion of NME6 [39,42], while depletion of NME6 seems to have less drastic effects on RCC1L [38]. In conclusion, while NME6 monomers are inactive, they gain NDPK activity through binding to RCC1L (Figure 2b). The formation of such complexes with an NME-unrelated protein to achieve kinase proficiency is unique and may represent a novel paradigm within the NME protein family.

The mechanism through which NME6 gains NDPK proficiency within the complex is still under investigation. One could hypothesize that the NDPK activity of a monomer requires stabilizing interactions with a partner, typically with other NME monomers within a hexamer. In the case of NME6, these interactions would be provided by RCC1L. Alternatively, it was proposed that RCC1L could be directly involved in NDPK activity since RCC1L seems to have autonomous ATP hydrolysis activity [39]. As proposed by the authors, this activity could provide the phosphate that is transferred to the active site histidine of NME6. However, the conserved active site of all NDPK-active NMEs requires fixation of ATP within the catalytic cleft for successful phosphotransfer to histidine. Therefore, the utilization of ATP by RCC1L may serve additional, as-yet-undiscovered functions (Figure 2b).

### 4.3. Role of NME6 in Pyrimidine Synthesis for mtDNA Transcription and Replication

The recent progress in NME6 biology has been primarily driven by large-scale, non-biased CRISPR/Cas9 screens targeting essential genes involved in mitochondrial ribonucleotide supply [38] or OXPHOS complex assembly [39]. These screens identified *nme6* as a top hit and demonstrated an essential role of NME6 NDPK activity.

Deletion of NME6 expression in various cellular systems decreased mitochondrial transcripts and reduced the levels of several mtDNA-encoded subunits in the respiratory complexes [38,39]. Notably, the loss of mtRNAs correlated with the distance from the heavy-strand mtDNA promoter, suggesting a depletion of local NTP levels with subsequent transcription. This led to a dysfunctional respiratory chain, causing impaired mitochondrial respiration, cell growth, and proliferation (Table 1). Such an effect was particularly prominent in media with low glucose or a non-fermentable carbon source like galactose. In all cases tested, the observed phenotypes could be rescued by the expression of wild-type NME6, but not of the NME6 kinase-inactive mutant, unequivocally demonstrating that the impaired mitochondrial NTP biosynthesis by NME6 is responsible for this phenotype. Interestingly, mtDNA replication, which necessitates dNTP nucleotides, remained unaffected in NME6 knock-out cells.

**Figure 3:**
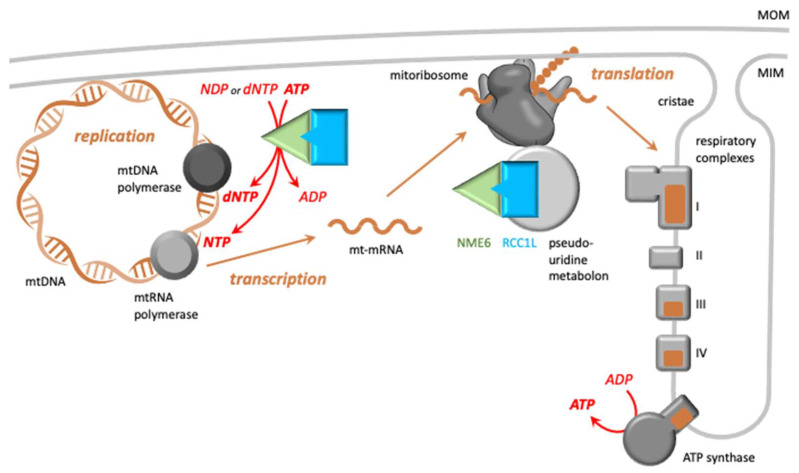
Functions of the NME6/RCC1L complex in mitochondrial gene expression. **Left part**: Putative NME6/RCC1L heterodimers close to or associated with the mitochondrial inner membrane (MIM) [37] and located in mitochondrial RNA granules are involved in replication and transcription of mtDNA by local supply of (d)NTPs, necessary for DNA and mtRNA synthesis by the respective polymerases. NME6 is essential for the mitochondrial salvage pathway to generate pyrimidine nucleotides, in particular CTP, and thus mtRNA synthesis [38,39]. Import from the cytosol seems to be sufficient for purine NTPs and most dNTPs. **Right part:** Altered mt-transcript levels will affect the encoded subunits (brown) within the respiratory complexes (grey), dysregulate their assembly, and hinder supercomplex formation. Further, interaction with RCC1L is not only essential for NDP kinase activity but could also have other, yet undefined, functions. This interaction allows NME to become part of the pseudouridine metabolon [42], localizing to mt-ribosomes. There, NME6 could contribute to mitoribosome function at different levels [39]. mtDNA and mitoribosomes are both associated with the mitochondrial inner membrane, where newly synthesized membrane proteins can be directly inserted. Color code: green/cyan, NME6/RCC1L complex (as in Figure 2); brown, mitochondrial DNA, mRNA, and mtDNA-encoded subunits of the respiratory chain. Abbr.: MOM, mitochondrial outer membrane; MIM, mitochondrial inner membrane.

Both mtRNA synthesis and mtDNA replication within the mitochondrial matrix require a continuous supply of NTPs (ribonucleotides) and dNTPs (deoxynucleotides), respectively (Figure 3, left part). The majority of these nucleotides are generated through de novo synthesis or the salvage pathway in the cytosol, followed by mitochondrial import of (d)NTPs or their direct precursors ((d)NDPs, (d)NMPs, nucleosides) via VDAC and different nucleotide transporters in the outer and inner mitochondrial membranes, respectively [55]. In addition, and particularly in rapidly proliferating cells, mitochondria have to synthesize a part of the required (d)NTPs via the mitochondrial salvage pathway from (d)NTP breakdown products. The final step of this salvage pathway is catalyzed by NMEs in the matrix, specifically NME4 and NME6.

A comprehensive analysis of mitochondrial nucleotide metabolism in NME6-deficient cells, without or with NME6 reexpression, revealed that only NME6, not NME4, plays an essential role for the nucleotide salvage pathway, specifically for pyrimidine NTPs (CTP, TTP, and UTP), not the purine NTPs or (d)NTPs [38]. The mitochondrial import of pyrimidine NTPs is insufficient for sustaining mitochondrial transcription, thus requiring the presence of NME6. Conversely, NME6 is dispensable for matrix dNTPs, as the demand can be met by importing dNTPs from the cytosol. Only if the corresponding mitochondrial transporters are depleted can NME6 also become essential for dNTP and mtDNA synthesis. Nonetheless, a specific reduction in matrix dCTP levels was already observed in NME6-deficient cells, but without affecting mtDNA replication [38]. This decrease is probably attributable to the consumption of dCTP in CTP-dependent processes, such as the synthesis of certain lipids, under conditions of CTP depletion [55].

The supplementation of media with NTPs or nucleoside precursors, but not with dNTPs, normalized CTP and mtRNA levels, confirming NME6’s primary role in maintaining the appropriate matrix NTP pools for transcription (Table 1). However, such supplementation did not completely correct the growth deficit, indicating potential NTP-pool-independent functions. This is also supported by NME overexpression, which led to a similar phenotype as NME-KO, namely impaired respiration and reduced levels of some mtDNA-encoded mitochondrial subunits of the respiratory chain [37]. It is thus plausible that NME6 has additional functions, likely dependent on its interaction partner RCC1L, a protein involved in mitoribosome assembly and function [54].

### 4.4. The Putative Role of NME6-RCC1L in Mitoribosome Function

RCC1L was identified in a genome-wide CRISPR screen for genes essential for oxidative phosphorylation [50]. It emerged as part of a functional assembly of six proteins (RCC1L, FASTKD2, NGRN, RPUSD3, RPUSD4, TRUB2) that are necessary for maintaining 16S mt-rRNA levels and mitochondrial translation (Figure 3, right part). They were also found as part of a functional module involved in RNA pseudouridylation within mitochondrial RNA granules [42]. NME6 was specifically mapped to this pseudouridylation module in large-scale mitochondrial proximity interaction mapping [56]. While RNA pseudouridylation is tightly regulated and may impact mtRNA stability and translation, its precise role within mitochondria remains incompletely understood. Proteins in the pseudouridylation module do not seem to form stable complexes but rather interact transiently, and only individually may they engage in more stable complexes [42], such as NME6-RCC1L [37].

More recently, three isoforms of RCC1L were identified, called RCC1L^V1^, RCC1L^V2^, and RCC1L^V3^ [57]. All three localize to the mitochondrial matrix, where they interact with the mitochondrial inner membrane. Further, RCC1L^V1^, the most abundant, and RCC1L^V3^ interact with the large and small mitoribosomal subunits, respectively, and were shown to participate in their assembly [57]. Notably, a recent RCC1L-KO in dopaminergic neurons has demonstrated RCC1L as essential for maintaining mitochondrial structure and function [58].

Both overexpression and depletion of RCC1L^V1^ and RCC1L^V3^ impaired mitoribosome biogenesis, including decreased levels of the corresponding mt-ribosomal subunits and 16S mt-rRNA [57]. This is reminiscent of observations with NME6, where both overexpression and depletion impaired mitochondrial respiration [37,38,39]. These findings suggest that NME6 and RCC1L interact not only structurally but also functionally, and that tight regulation of their expression levels is necessary for correct function. While it has been demonstrated that stability and NDPK activity of NME6 rely on its interaction with RCC1L [39], NME6 could also affect RCC1L functions with respect to 16S mt-rRNA levels, RNA pseudouridylation, or mitoribosome assembly. For instance, RCC1L’s impact on pseudouridylation might involve its putative GDP/GTP nucleotide exchange factor activity [57,59], which could be locally fueled with GTP by NME6. Although NME6 is not essential for mitoribosome assembly [38], its depletion leads to assembly defects, especially in the small mitoribosomal subunit, and to modifications in pseudouridylation at certain mt-mRNA sites [39]. Notably, these alterations were rescued by the reexpression of wild-type NME6, demonstrating the involvement of NDPK activity.

In summary, within NME6-RCC1L complexes, NME6 function extends beyond the global supply of (d)NTPs. However, this topic definitively requires further, more detailed investigation.

## 5. Perspectives

Recent progress in understanding NME6 biology has resolved many questions in regard to its canonical function in (d)NTP supply and the absence of NME6 hexamers. The novel mechanism by which NME monomers are stabilized and kept in a NDPK active form by interaction with a non-NME protein partner may represent a new paradigm that might extend to other group II NME proteins sharing a well-conserved NDPK domain.

However, these recent studies have also given rise to several intriguing questions. Why does a cell require two NDPK-active NMEs in the mitochondrial matrix? The essential role of NME6 for pyrimidine NTP supply and mitoribosome function, despite the presence of NME4 within the same compartment, argues for suborganellar metabolic compartmentalization with distinct and highly localized roles for these two NME isoforms. However, evidence for such roles is only emerging, e.g., with NME6 co-localizing or cosedimenting with mtDNA nucleoids, mtRNA granules, and mitoribosomes [38]. Is this localization inherent to NME6, or is it recruited by RCC1L into these sites? Further, does this localization only influence local (d)NTP supply, or does it serve a broader purpose in spatial control of translation? Regarding the matrix NME4, it is also important to recognize that this population, at least in mammals, may be relatively minor compared with the predominant IMS-localized NME4. Further exploration of the matrix localization and abundance of NME4 and NME6 is necessary to elucidate their specific roles in metabolic compartmentation.

The fundamental new paradigm of NME6 stably interacting with a non-NME protein also raises new questions. They first concern the structural aspects: Is the stoichiometry of the basic complex structure indeed a heterodimer, and is all NME6 then recruited into such complexes with RCC1L, as suggested by the strong depletion of NME6 in RCC1L-KO cells? How does RCC1L alter the structure of NME6 to render it NDPK active? Functionally, by binding with RCC1L, does NME6 also modulate the functions of its binding partner, as discussed above? To address these questions, a deeper understanding of the molecular structure and mechanisms of RCC1L-NME6 complexes is required. How precisely does RCC1L regulate 16S mRNA stability and ribosome assembly? Is this regulated through altered RNA pseudouridylation, a poorly understood process in mitochondria, with RCC1L acting as a GDP/GTP exchange factor on ribosome GTPases and pseudouridylation proteins as proposed earlier [57,60]? Such guanine-nucleotide exchange activity was reported for RCC1L located in the IMS [59] and is known for other members of the RCC family, like RCC1 [61]. In this case, NME6 could directly fuel RCC1L with GTP. Alternatively, the ATP hydrolysis activity of RCC1L could play a role [39], albeit distinct from the NME6 NDPK function, as discussed above. All these unresolved questions await further investigation.

Finally, based on the recent paradigm-shifting insights into the structure and function of NME6, its role in human disease merits further (re)evaluation. There is emerging evidence for a predominant role of NME6 in matrix (d)NTP supply and mitochondrial function in rapidly proliferating cancer cells. Upregulated NME6 has been associated with progression and unfavorable outcomes in some cancers [34], potentially positioning NME6 as a promising diagnostic or prognostic marker or even as a target for anticancer therapies. Given the ubiquitous role of inflammation in diverse pathologies, further investigation into the involvement of all mitochondrial NMEs (NME3, 4, and 6) in inflammasome activation [35] is warranted, especially considering the current lack of clarity on the mechanistic basis. While mitochondria are known as a hub for inflammasome activation, unraveling how exactly all three NMEs favor this process and if this occurs through similar or distinct mechanisms remains to be established.

Further reviews on NME proteins appear in a special issue of the Int. J. Mol. Biol. in 2024.

## Figures and Tables

**Table 1:** Phenotypes of NME knock-out or overexpression and rescue with NME variants or nucleosides/nucleotides.

Genotype ^1^		Supplement	Phenotype (versus WT Phenotype) ^2^					
*nme6*	*SLC25A33* *SLC25A36*	Nucleotides or Nucleosides	Proliferation	Respiration, RC Subunits	mtDNA	mtRNA	Mitochondrial Pyrimidine Nucleotides	
KO			↓ ^5^	↓ ^6^	=	↓ ^6^	↓ CTP, dCTP	
							↑ CMP, CDP, UMP, UDP	
KO	KO		↓ ^5^		↓			
KO + WT ^3^			=	=	=	=	=	
KO + KI ^3^				↓	↓			
KO		NTPs		=		=		
KO		dNTPs				↓		
KO		nucleoside mix ^4^		=		=		
KO		uridine (+ cytidine)	(=)	=		=	=	
OE			=	↓				

^1^ Genotypes: WT, wild-type; KO, knock-out; KI, kinase-inactive H137N/A mutants; OE, overexpression; KO data from [38,39], OE data from [37]. ^2^ Rating: ↓, reduced levels; =, unchanged/restored levels; (=) partially restored levels; ↑, increased levels. ^3^ Rescue experiment by re-expressing NME6 wild-type or NME6 kinase-inactive H137N mutant. ^4^ Complete mix with pyrimidines (cytidine, uridine, and thymidine) and purines (guanosine, adenosine). ^5^ Strong decrease or lethal in media requiring oxidative phosphorylation (galactose, human plasma-like medium [38]). ^6^ Differential effects on respiratory chain complexes (mainly CI and CIII are downregulated, CIV increased) and mtRNAs (mostly downregulated, but early transcribed genes close to the heavy strain promoter are upregulated, including CIV-COX1 [39]. Abbr.: SLC25A33, SLC25A36, mitochondrial pyrimidine (deoxy)ribonucleotide transporters; RC, respiratory chain. Cell lines used in these studies: MDA-MB-231T [37]; HeLa, HepG2, HLE, Huh6 [38]; K562, HEK293T, and U2OS [39].

## Data Availability

The immunoblot dataset is available from the corresponding authors on reasonable request.
